# Tanshinone IIA Attenuates Diabetic Peripheral Neuropathic Pain in Experimental Rats via Inhibiting Inflammation

**DOI:** 10.1155/2018/2789847

**Published:** 2018-03-11

**Authors:** Baojian Zhang, Yanbing Yu, Gele Aori, Qi Wang, Dawei Kong, Wenqiang Yang, Zhuangli Guo, Li Zhang

**Affiliations:** ^1^Peking University China-Japan Friendship School of Clinical Medicine, Beijing 100029, China; ^2^Department of Neurosurgery, China-Japan Friendship Hospital, Beijing 100029, China; ^3^Rehabilitation Department, The Affiliated Hospital of Qingdao University, Qingdao 266071, China

## Abstract

Diabetic peripheral neuropathic pain (DPNP) is a common and intractable complication of diabetes. Conventional therapies are always not ideal; development of novel drugs is still needed to achieve better pain relief. Recent evidences have demonstrated that inflammation is involved in the onset and maintenance of DPNP. The anti-inflammatory property of Tanshinone IIA (TIIA) makes it a promising candidate to block or alter the pain perception. This study was conducted to investigate whether TIIA could attenuate DPNP in streptozotocin- (STZ-) induced rats model and its potential mechanisms. TIIA was administered to STZ-induced diabetic rats at the dose of 40 mg/kg once a day for 3 weeks. The effects of TIIA on thermal hyperalgesia and mechanical allodynia were investigated using behavioral tests. The mRNA level and expression of interleukin- (IL-) 1*β*, interleukin- (IL-) 6, tumor necrosis factor- (TNF-) *α*, and interleukin- (IL-) 10 in the fourth to sixth segments of the dorsal root ganglion (L4–6 DRG) were detected by quantitative real-time PCR (qPCR) and Western blot. TIIA treatment significantly attenuated mechanical allodynia and thermal hyperalgesia in diabetic rats. In addition, the expression of the proinflammatory cytokines IL-1*β*, IL-6, and TNF-*α* was inhibited, and the level of the anti-inflammatory cytokine IL-10 was increased by TIIA. This study demonstrated that TIIA has significant antiallodynic and antihyperalgesic effects in a rat model of STZ-induced DPNP, and the effect may be associated with its anti-inflammation property.

## 1. Introduction

The prevalence of DPNP ranges from 20% to 24% in patients with diabetes and from 40% to 50% in those with diabetic neuropathies [1, 2]. DPNP is characterized by spontaneous pain, hyperalgesia, and allodynia, some of which even progressed to hypoalgesia at advanced stage, which often leads to sleep disorder and depression and passively affects patients' health-related quality of life [3]. In addition to the negative impact on individuals, it also leads to heavy social and economic burden [4]. The mechanism underlining DPNP is still unclear. The existing treatment for DPNP is mainly focusing on glycemic control and lifestyle changes. Currently available drugs such as antidepressants, sodium and calcium channel blockers, and opioids are often ineffective and complicated by adverse events [5]. Therefore, it is imperative to search effective agents for the distressing complication of diabetes.

*Salvia miltiorrhiza* Bunge (Danshen in Chinese) is one of the most famous Chinese traditional herbs with more than 1000 years of clinical applications [6]. The biological activities of Danshen have been widely investigated, and more than 100 pure compounds have been isolated from it, which are classified as hydrophilic phenolics and lipophilic quinines [6]. TIIA is one of the major diterpenoids derived from Danshen, which exhibited diverse pharmacological activities, such as antioxidative [7], anti-inflammation [8], and anticancer [6, 9]. TIIA has been widely used for the treatment of a variety of diseases, including atherosclerosis [10], cerebral infarction [11], and spinal cord injury [12]. In diabetic animal models, TIIA not only protects against diabetic nephropathy [13], but also has protective effects on impaired motor and sensory nerve functions in diabetic neuropathy [14]. Previous studies have shown that TIIA has significant antinociceptive effects in a complete Freund's adjuvant- (CFA-) induced inflammation pain model as well as in bone cancer pain model through suppression of the transcription of proinflammatory cytokine genes [15, 16]. However, it remains unclear whether TIIA exerts antinociceptive effects in a STZ-induced DPNP model. Therefore, the present study was designed to explore the therapeutic action of TIIA in a rat model of DPNP.

## 2. Materials and Methods

### 2.1. Animals

A total of 40 male Sprague-Dawley (SD) rats were purchased from Vital River Company (Beijing, China), weighing between 200 and 220 g, which were allocated into 3 groups. The animals were housed in the SPF room under controlled laboratory conditions (temperature, 23 ± 2°C; relative humidity, 40–60%, 12 h light/dark cycle) and allowed free access to food and water. All experiments were carried out between 09:00 and 17:00 h. Due to polyuria, animal bedding was changed daily. The animal study was approved by the Institutional Animal Ethics Committee of the Institute of Clinical Medicine of China-Japan Friendship Hospital. As some suffering might result from the experiments, the International Association for the Study of Pain (IASP) Committee for Research and Ethical Issues guidelines were followed [17].

### 2.2. Drugs and Reagents

Tanshinone IIA (purity > 99%) was obtained from Chengdu de Stewart Biological Technology Co., Ltd. (Sichuan, China). Streptozotocin (STZ) was purchased from Sigma (St. Louis, MO, USA). The following primary antibodies (Abcam, MA, UK) were used in the Western blotting analyses: TNF-*α* (ab6671), IL-1*β* (ab2105), IL-6 (ab9324), and IL-10 (ab33471); antibody against *β*-actin (sc-47778) was purchased from Santa Cruz Biotechnology (Santa Cruz, CA, USA). Enhanced chemiluminescence (ECL) detection reagents were obtained from HuaXingbio Centre of Biotechnology (Beijing, China); bicinchoninic acid (BCA) protein assay kits and RIPA lysis buffer were obtained from Beyotime Institute of Biotechnology (Haimen, China).

### 2.3. Experimental Protocol

After one week of adaptation, the diabetes was induced in overnight fasted animals by a single intraperitoneal injection of STZ (60 mg/kg, freshly prepared in 0.1 mol/L citrate buffer, pH 4.5). Age and body weight matched control rats were injected with the equal amount of vehicle (0.1 mol/L citrate buffer, pH 4.5). Three days after STZ injection, the blood glucose level was measured in a tail vein blood sample. Rats with a blood glucose level greater than 16.7 mmol/L were included in the study. Rats in each group were treated as follows: control group (*n* = 12): normal animals treated with vehicle (distilled water intraperitoneally); DM group (*n* = 12): diabetic animals treated with vehicle (distilled water intraperitoneally); DM + TIIA group (*n* = 12): diabetic animals treated with TIIA 50 mg/kg. Treatment was started 3 weeks after STZ injection and lasted for 3 weeks [18]. During this period, diabetic animals showed symptoms of polydipsia, polyuria, polyphagia, and weight loss [19]. The TIIA powder was dissolved in distilled water (concentration 0.5%) and administered to the rats by intraperitoneal injection daily; the dose was determined based on previous literatures [14, 20].

Blood glucose levels were monitored at intervals during the experiment. Behavioral assessments like thermal hyperalgesia and mechanical allodynia were performed on 0th, 3rd, 4th, 5th, and 6th week of the study. At the end of the 6th week, immediately after behavioral assessment, rats in all the groups were anaesthetized with pentobarbital sodium (40 mg/kg). Under dissecting microscope, DRG at L4–L6 levels were removed and immediately frozen in liquid nitrogen and stored at −80°C until biochemical analysis. Then the rats were sacrificed by cervical dislocation.

### 2.4. Behavioral Tests

#### 2.4.1. Assessment of Allodynia

Allodynia was assessed by determination of the paw withdrawal threshold by mean of von Frey test. The procedure was described and previously validated by Chaplan et al. [21]. In brief, rats were placed in a testing cage with a wire mesh bottom; behavioral adaptation was allowed for 15–20 min, until the major grooming activities and cage exploration ceased. Von Frey hairs (1.4, 2, 4, 6, 8, 10, 15, and 26 g) were applied to the middle of the plantar surface of the hind paw, for a maximum of 8 s to elicit a paw withdrawal thresholds. Abrupt paw withdrawal, licking, and shaking were considered to be positive responses. The same procedure was repeated three times on the same paw, with at least 5-minute interval between two successive procedures. The mean of the three values was considered as paw withdrawal threshold for each rat.

#### 2.4.2. Assessment of Hyperalgesia

Hyperalgesia was assessed by measuring the paw withdrawal latency to radiant heat using the Hargreaves method [22]. Briefly, rats were placed in an organic glass box without restraint; after a 15–20 min habituation period the plantar surface of the paw was exposed to the infrared radiant heat (model 390, IITC Life Science, Woodland Hills, CA, USA) through the glass floor. The paw withdrawal latency was defined as the time from onset of the radiant heat to the withdrawal of the rat hind paw. The heat source was adjusted to a mean baseline value about 10–12 seconds and a cutoff time of 20 seconds to prevent tissue damage. Testing was performed 4 times on the same paw with a 5-minute interval, and the mean of the last three values was considered as the thermal withdrawal latency.

### 2.5. Quantitative Real-Time PCR (qPCR)

Thawed L4–L6 DRG were homogenized in 1 mL of Trizol reagent (Beyotime, Haimen, China); total RNA was isolated according to the manufacturer's instructions. The purity of RNA was quantified by spectrophotometer (A260/280). cDNA was synthesized from total RNA using a PrimeScript™ RT reagent kit with gDNA Eraser (Takara Bio, Otsu Shiga, Japan). The reverse transcription reaction was performed under the following conditions: (i) 37°C for 15 min and (ii) 85°C for 5 min, followed by storing at 4°C. qPCR reactions were carried out on a ABI 7500 real-time PCR System (Applied Biosystems, USA) with SYBR Green I PCR mix kit (Applied Biosystems). Each reaction consisted of a volume of 20 *μ*l sample (2 *μ*l cDNA, 10 *μ*l 2x SYBR Green mixtures, 0.4 *μ*l of each primer, and 7.2 *μ*l of nuclease-free water). The protocol consisted of an initial denaturation at 95°C for 2 min, followed by 40 cycles of denaturing at 95°C for 30 sec, annealing at 60°C for 30 sec, and extension at 72°C for 30 sec. Melting curves were generated at the end of every run to ensure product uniformity. Each sample was performed in triplicate, and the results were averaged. GAPDH was used as an internal control transcript. Relative changes in mRNA levels were determined by the 2^−ΔΔCT^ method as previously described. Primers sequences used in real-time PCR are summarized in Table 1.

### 2.6. Western Blot Analysis

The frozen L4–L6 DRG was thawed and homogenized in ice-cold RIPA lysis buffer (Beyotime) containing a protease inhibitor cocktail (Roche). Then the homogenized tissues were centrifuged at 12,000 rpm for 15 min at 4°C, and the resulting supernatant was collected. Protein concentrations of the extracts were measured by BCA assay. Protein samples (50 ug) were denatured at 95°C for 5 minutes, separated by SDS-PAGE, and then transferred onto PVDF membranes (Millipore). The membranes were blocked with 5% nonfat milk for 1 h at room temperature and then incubated with the primary antibodies: TNF-*α* (1 : 1000; ab6671), IL-1*β* (1 : 1000; ab2105), IL-6 (1 : 1000; ab9324), IL-10 (1 : 1000; ab33471), and *β*-actin (1 : 10000; sc-47778) at 4°C overnight, followed by washes with TBST. Finally, the membranes were incubated with HRP-conjugated secondary antibody for 1 h at room temperature. The signal was detected with Super ECL kit and X-ray film. Specific bands were evaluated by apparent molecular sizes. The intensity of the selected bands was quantified by ImageJ software and standardized with the *β*-actin density.

### 2.7. Statistical Analysis

Results were analyzed by SPSS (Ver19.0). The significant differences were analyzed by one-way analysis of variances (ANOVA), followed by least significant difference (LSD) test. All results were presented as mean ± SEM, and a *P* value < 0.05 was considered statistically different. 

## 3. Results

### 3.1. Effects of TIIA on Body Weight and Blood Glucose Level

**Figure 1:**
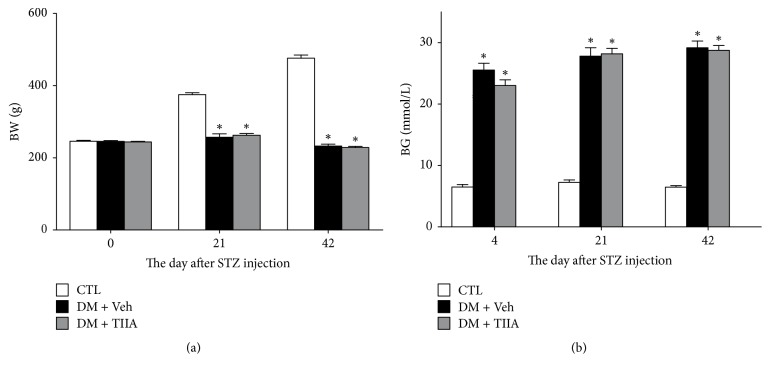
*The effect of Tanshinone IIA on body weight and blood glucose in STZ-induced diabetic rats*. (a) Body weight (BW); (b) blood glucose (BG). CTL, normal rats; DM + Veh, DM rats treated with vehicle; DM + TIIA, DM rats treated with Tanshinone IIA. Results are expressed as means ± SEM (*n* = 12/each group, ^*∗*^*P* < 0.01 versus CTL group).

By the 3rd day after STZ injection, 85.7% of the rats developed hyperglycemia (*n* = 24); their blood glucose levels were statistically higher than the control rats (*P* < 0.01), which did not significantly change during the experiment. There was a marked weight loss of STZ-induced diabetic rats as compared with control rats (*P* < 0.05). TIIA treatment did not alter the body weight and the blood glucose level (data are shown in Figure 1).

### 3.2. Effects of TIIA on Thermal Hyperalgesia and Mechanical Allodynia

**Figure 2:**
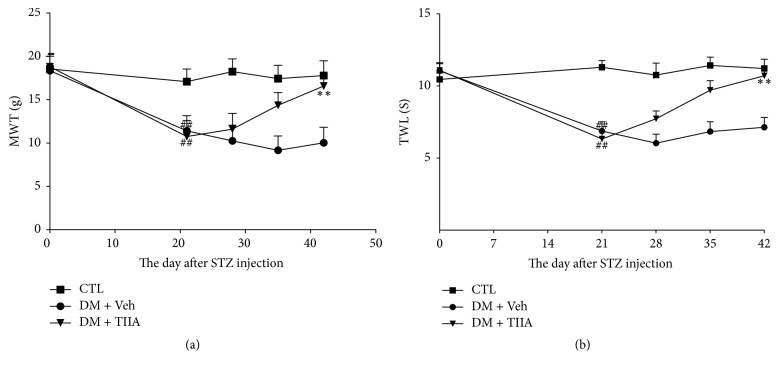
*The effect of Tanshinone IIA on MWT and TWL in STZ-induced diabetic rats*. (a) Mechanical paw withdrawal threshold (MWT); (b) thermal paw withdraw latency (TWL). CTL, normal rats; DM + Veh, DM rats treated with vehicle; DM + TIIA, DM rats treated with Tanshinone IIA. Results are expressed as means ± SEM (*n* = 12/each group, ^##^*P* < 0.01 versus CTL group, ^*∗∗*^*P* < 0.01 versus DM + Veh group).

At the end of the 3rd week, mechanical paw withdrawal threshold and thermal paw withdrawal latency were significantly lower in DM and DM + TIIA groups than in control group (*P* < 0.05). TIIA treatment for 3 weeks increased the paw withdrawal threshold and paw withdrawal latency in DM + TIIA group compared with DM group (*P* < 0.05) (Figure 2).

### 3.3. Effects of TIIA on mRNA Expression of Inflammatory Cytokines (IL-1*β*, IL-6, TNF-*α*, and IL-10) in the DRG (L4–L6)

**Figure 3:**
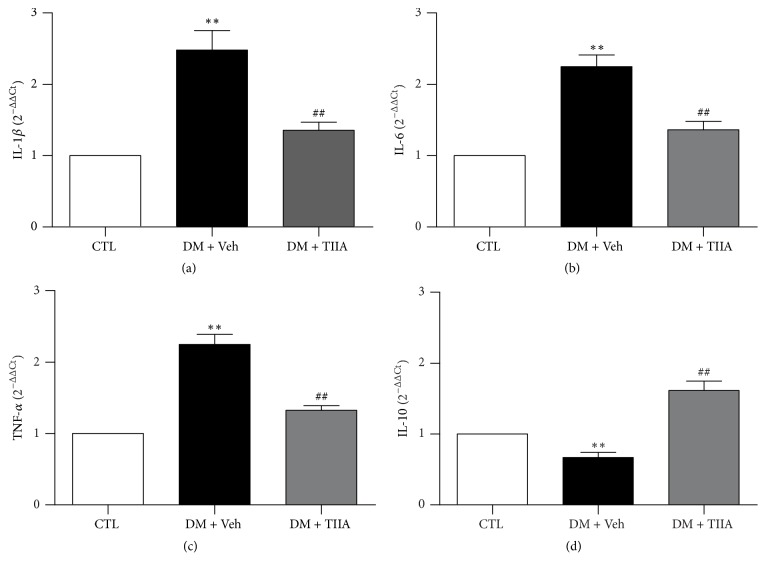
*The effect of Tanshinone IIA on mRNA level of IL-1β, IL-6, TNF-α, and IL-10 in the DRG from experiment rats*. The mRNA levels of IL-1*β*, IL-6, TNF-*α*, and IL-10 were analyzed with qPCR. CTL, normal rats; DM + Veh, DM rats treated with vehicle; DM + TIIA, DM rats treated with Tanshinone IIA (*n* = 6 for each group;, ^*∗∗*^*P* < 0.01 versus CTL group, ^##^*P* < 0.01 versus DM + Veh group).

To understand the molecular mechanism underlying the function of TIIA, qPCR was used to determine the mRNA levels of IL-1*β*, IL-6, TNF-*α*, and IL-10 in DRG. At the end of the study, in DM group, the mRNA levels of IL-1*β*, IL-6, and TNF-*α* in DRG were significantly higher (*P* < 0.05) and IL-10 was lower than in the control group (*P* < 0.05). 3 weeks of treatment with TIIA reduced the mRNA level of the proinflammatory cytokines (IL-1*β*, IL-6, and TNF-*α*) and elevated the anti-inflammation cytokine (IL-10) (Figure 3).

### 3.4. Effects of TIIA on Protein Expression of Inflammatory Cytokines (IL-1*β*, IL-6, TNF-*α*, and IL-10) in DRG (L4–L6)

**Figure 4:**
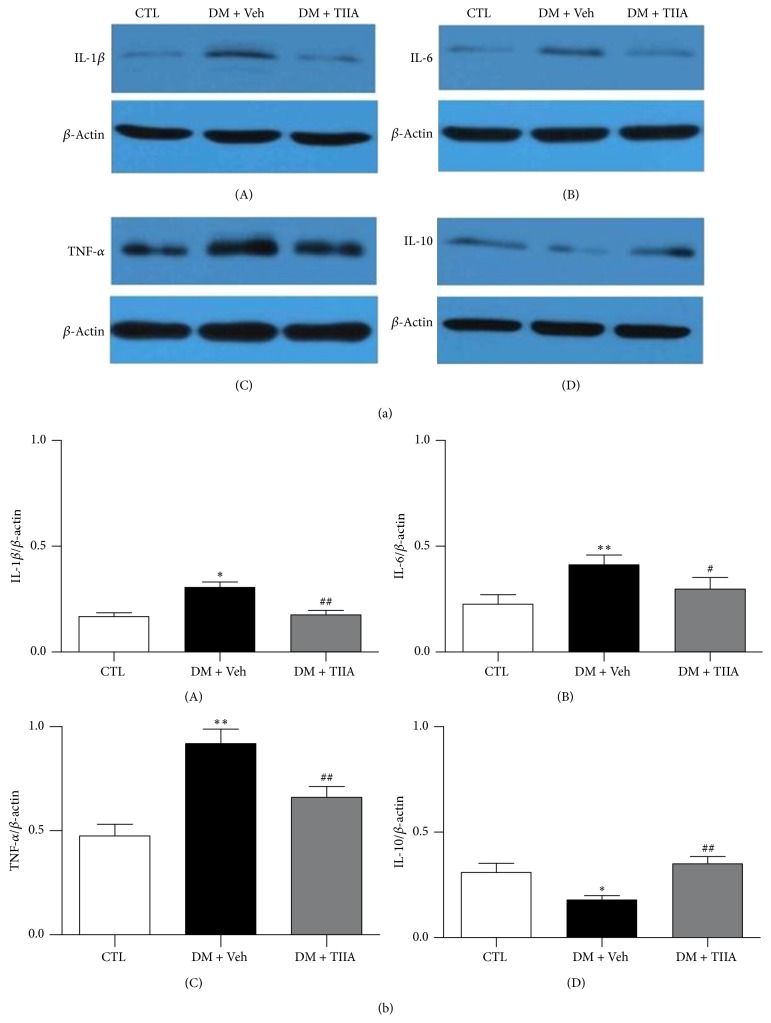
*The effect of Tanshinone IIA on the protein expression of IL-1β, IL-6, TNF-α, and IL-10 in the DRG from experiment rats*. The protein levels of IL-1*β*, IL-6, TNF-*α*, and IL-10 were determined by Western blot. (a) Western blot results of the IL-1*β*, IL-6, TNF-*α*, and IL-10. (b) The target protein bands were densitometrically analyzed after normalization to *β*-actin. Each experiment was repeated three times and similar results were obtained. CTL, normal rats; DM + Veh, DM rats treated with vehicle; DM + TIIA, DM rats treated with Tanshinone IIA. (*n* = 6 for each group; ^*∗*^*P* < 0.05, ^*∗∗*^*P* < 0.01 versus CTL group; ^#^*P* < 0.05, ^##^*P* < 0.01 versus DM + Veh group).

The protein levels of IL-1*β*, IL-6, and TNF-*α* were increased and IL-10 was decreased in DM group compared with control rats. As shown in Figure 4, 3 weeks of treatment with TIIA led to significantly lower expression of IL-1*β*, IL-6, and TNF-*α* and significant upregulation of IL-10.

## 4. Discussion

In this study, we found that TIIA treatment attenuated mechanical allodynia and thermal hyperalgesia in the rats with diabetes. In addition, our data confirmed that the pain behavior coincided with the expression changes of inflammatory cytokines (TNF-*α*, IL-1*β*, IL-6, and IL-10) in rats with diabetes induced by STZ. Moreover, TIIA treatment reduced the proinflammatory cytokines level (TNF-*α*, IL-1*β*, and IL-6) and elevated anti-inflammatory cytokine level (IL-10) in DRG of STZ-induced diabetic rats. All the findings suggest an antiallodynic and antihyperalgesic capability of TIIA against inflammatory response in STZ-induced diabetic rats.

In recent years, STZ-induced diabetic rat has been widely used in DPN studies [19, 23]. The present study showed that after 3 weeks of STZ injection diabetic rats manifested decreased paw withdrawal threshold and paw withdrawal latency, indicating the appearance of mechanical allodynia and thermal hyperalgesia. These findings are concordant with observations in some previous studies [24, 25]. However, some other reports demonstrated a thermal hypoalgesia in diabetic animals [19, 23, 26]. We speculate that the discrepancy may be related to the following factors: diabetic duration (early or later stages); severity of the diabetic state; different behavioral test methods and kinds of animals used in experimental studies.

It is well accepted that most of the diabetic complications, including DPNP, are caused by protracted hyperglycemia. Our study showed that 3 weeks of treatment with TIIA significantly attenuated the mechanical allodynia and thermal hyperalgesia but did not influence the blood glucose level. Some previous studies have also reported many other compounds which have beneficial effects on diabetic complications, including neuropathy and nephropathy without any influence on STZ-induced hyperglycemia and body weight loss [27, 28]. Furthermore, in both human and animal studies, conventional DM therapy and even enhanced blood glucose control have failed to prevent diabetic neuropathy [29]. These data indicate that the therapeutic effects of TIIA on DPNP have no relationship with blood glucose level.

The pathogenesis of diabetic neuropathy is complicated and involves multiple pathways. In the last decade, studies have demonstrated that the pathogenesis of neuropathic pain is associated with the interactions between neurons, inflammatory immune cells, and immune cell-derived inflammatory cytokines [30]. Metabolic changes induced by hyperglycemia lead to increased secretion of inflammatory cytokines by both activated resident immune cells and recruited immune cells, which induces biochemical changes and nerve damage, resulting in pain-related symptoms eventually [25, 29]. Increasing evidences indicate that inflammatory cytokines play key roles in the development and maintenance of neuropathic pain. Previous study has shown that patients with painful neuropathy have increased mRNA and protein levels of IL-2 and TNF-*α* and decreased levels of IL-4 and IL-10 [31]. In animal models, altered pain behaviors are associated with elevated TNF-*α* levels [32], and intraneural application of proinflammatory cytokines IL-1*β* and TNF-*α* induces behavioral signs associated with pain [33]. In the present study, our data showed that the increased proinflammatory cytokines (IL-1*β*, TNF-*α*, and IL-6) and decreased anti-inflammatory cytokine (IL-10) in the DRG may be related to the allodynia and hyperalgesia in STZ-induced diabetic rats.

Modulating the cytokines production by downregulating proinflammatory cytokines and/or upregulating anti-inflammatory cytokines and restoring the balance of pro-/anti-inflammatory cytokines in the nervous system have been considered as treatment strategies for neuropathic pain [34]. Intrathecal administration of IL-1*β* and TNF*α* antagonists as well as IL-6 neutralizing antibody attenuates pain behaviors in neuropathic models [35–37]. HSV mediated local expression of IL10 in the DRG inhibits the development of painful neuropathy in diabetic rats [38]. However, it is considered that IL-1*β*, TNF-*α*, and IL-6 induce the production of each other through positive feedback, acting synergistically to amplify the inflammatory signals [39], which can of course lead to a vicious circle of chronic inflammation if not adequately suppressed, so it is difficult to achieve satisfactory improvement of pain symptoms by modulating just one single cytokine. Unlike the cytokine antagonist by blocking only one specific inflammatory cytokine, herbal plant extracts affect productions of multiple cytokines, reduce excessive inflammatory responses, and cause relatively little side effects. In animal models, genistein relieves neuropathic pain following nerve injury via downregulation of both IL-1*β* and IL-6 [40]; analgesic effect of coumarins is mediated by reducing spared nerve injury- (SNI-) induced upregulation of proinflammatory cytokines TNF-*α*, IL-1*β*, and IL-6 in damaged DRG [41]; bioactive fractions of annona reticulate bark attenuate painful diabetic neuropathy through decreasing the inflammatory cytokines (IL-1*β*, IL-6, and TNF-*α*) and increasing anti-inflammatory cytokine (IL-10) [42]. In line with the literature, in the present study, after 3 weeks of treatment with TIIA, mRNA and protein levels of IL-1*β*, IL-6, and TNF-*α* were reduced, IL-10 expression was elevated, and the mechanical allodynia and thermal hyperalgesia were alleviated simultaneously.

There are some limitations in the present study. We only focused on cytokines expressions in the DRG in our study. In others' experiments, the cytokines were also measured in the sciatic nerve, the DRG, and the spinal cord [40, 43–45]. However, the overall tendency of alterations of mRNA and protein levels was the same. Besides, hyperglycemia is known to induce oxidative stress which leads to nerve damage resulting in DPNP [46]. The antioxidant property of TIIA may also contribute to its antiallodynic and antihyperalgesic effects [7]. Further studies are needed to explore alternative mechanisms that underline the therapeutic effects of TIIA.

In conclusion, this study reveals that TIIA treatment alleviates mechanical allodynia and thermal hyperalgesia in a STZ-induced diabetic rat model. Furthermore, TIIA significantly modulates the expressions of several inflammatory cytokines in this model. Therefore, the antinociceptive activity of TIIA could be attributed, at least in part, to its anti-inflammatory properties. These findings suggest that TIIA might be a candidate medication for DPNP treatment. Further investigations in larger animals or even human are still needed.

## Figures and Tables

**Table 1:** Summary of the primer sequences for PCR analysis of the target genes.

Target gene	Forward	Reverse
IL-1*β*	CCTCTGCCAAGTCAGGTCTC	GAATGTGCCACGGTTTTCTT
TNF-*α*	GAGAGATTGGCTGCTGGAAC	TGGAGACCATGATGACCGTA
IL-6	CACAAGTCCGGAGAGGAGAC	CAGAATTGCCATTGCACAAC
IL-10	GTTGCCAAGCCTTGTCAGAAA	TTTCTGGGCCATGGTTCTCT
GAPDH	AATGTGTCCGTCGTGGATCTGA	GATGCCTGCTTCACCACCTTCT
